# Biodistribution and Adjuvant Effect of an Intranasal Vaccine Based on Chitosan Nanoparticles against Paracoccidioidomycosis

**DOI:** 10.3390/jof9020245

**Published:** 2023-02-12

**Authors:** Samuel Rodrigues Dos Santos Júnior, Filipe Vieira Barbalho, Joshua D. Nosanchuk, Andre Correa Amaral, Carlos Pelleschi Taborda

**Affiliations:** 1 Laboratory of Pathogenic Dimorphic Fungi, Department of Microbiology, Institute of Biomedical Sciences, University of São Paulo, São Paulo 05508000, Brazil; 2 Department of Medicine and Department of Microbiology and Immunology—The Bronx, Albert Einstein College of Medicine, New York, NY 10461, USA; 3 Laboratory of Nano&Biotechnology, Department of Biotechnology, Institute of Tropical Pathology and Public Health, Federal University of Goiás, Goiânia 74605050, Brazil; 4 Laboratory of Medical Mycology, School of Medicine/IMT/SP-LIM53, University of São Paulo, São Paulo 05403000, Brazil

**Keywords:** PCM, intranasal vaccine, chitosan nanoparticles, adjuvant nanoparticles, fluorescent nanoparticles

## Abstract

Paracoccidioidomycosis (PCM) is a fungal infection caused by the thermodimorphic *Paracoccidioides* sp. PCM mainly affects the lungs, but, if it is not contained by the immune response, the disease can spread systemically. An immune response derived predominantly from Th1 and Th17 T cell subsets facilitates the elimination of *Paracoccidioides* cells. In the present work, we evaluated the biodistribution of a prototype vaccine based on the immunodominant and protective *P. brasiliensis* P10 peptide within chitosan nanoparticles in BALB/c mice infected with *P. brasiliensis* strain 18 (Pb18). The generated fluorescent (FITC or Cy5.5) or non-fluorescent chitosan nanoparticles ranged in diameter from 230 to 350 nm, and both displayed a Z potential of +20 mV. Most chitosan nanoparticles were found in the upper airway, with smaller amounts localized in the trachea and lungs. The nanoparticles complexed or associated with the P10 peptide were able to reduce the fungal load, and the use of the chitosan nanoparticles reduced the necessary number of doses to achieve fungal reduction. Both vaccines were able to induce a Th1 and Th17 immune response. These data demonstrates that the chitosan P10 nanoparticles are an excellent candidate vaccine for the treatment of PCM.

## 1. Introduction

Paracoccidioidomycosis (PCM) is a mycosis that mainly affects the lungs but can lead to disseminated disease when the immune response is inadequate to control the spread of the fungus. PCM can be classified as a PCM infection and a PCM disease, in which PCM infection has no clinical manifestations and PCM disease is classified into two sub-forms: juvenile/acute/subacute or adult/chronic disease [1,2,3].

PCM is caused by thermodimorphic fungi of the genus *Paracoccidioides*, which is currently comprised by five known species: *P. brasiliensis, P. americana, P. restrepiensis, P. venezuelensis and P. lutzzi.* These fungi are found in almost every country in Latin America [4,5].

The effective control of PCM requires a Th1 and Th17 immune response with the production of IL-2, IL-12, IFN-γ, IL-17 and IL-23, which are responsible for stimulating phagocytosis and the formation of epithelioid granulomas [6,7]. In contrast, a Th2 response is prevalent in the most severe cases, usually in immunosuppressed patients [6,7].

The main drugs to treat PCM are polyene drugs (e.g., amphotericin B), sulfanilamide compounds (e.g., sulfadiazine), and azole drugs (e.g., itraconazole) [2,3,8]. Although these drugs are quite effective in treating PCM, they have numerous adverse effects, such as amphotericin B causing headaches, liver and kidney toxicity, and skin rashes [9].

A promising strategy to treat PCM is to use a therapeutic vaccine to generate a strong Th1 and Th17 immune response. One of the most researched candidate vaccines is based on the P10 peptide that has 15 amino acids (QTLIAHTLAIRYAN) derived from the immunodominant glycoprotein Gp 43 of *P. brasiliensis* [1,6,10,11]. Additionally, P10 peptide nanoparticles using polymers such as PLGA (poly(lactic-co-glycolic acid)) and chitosan can deliver a sustained release of the peptide resulting in significant protection from, and improvement of, PCM disease [12,13,14,15,16].

An important feature of chitosan nanoparticles is their ability to be mucoadhesive. This mucoadhesiveness promotes the attachment of nanoparticles to tissues, increasing their time of contact with cells and, potentially, leading to a better immune response [17,18,19,20,21]. For vaccine development, it is important to define the path taken by nanoparticles after administration, their location inside the cell, and which cells are responsible for their uptake, as this information facilitates subsequent improvements in the methods of nanoparticle delivery and provides insights into modifications to the particles that can vary the release of the P10 peptide [22,23,24,25,26].

When studying nanoparticles vaccines, several factors can affect the delivery, biodistribution and effect of the preparation. One of the most important factors is the complexation, or not, of the active molecule. This distinction can alter the time of processing of the vaccine, the presentation of the antigen and the lifetime of the antigen as well as the protection of the active molecule from degradation in the physiological environment of the live organism [22,23,24,25,26].

To evaluate the biodistribution, the necessity of complexation, adjuvant effect and the number of doses required to reduce the fungal load of the P. brasiliensis strain 18 (Pb18), we evaluated the administration of different chitosan nanoparticles vaccine formulations and dosing approaches.

## 2. Materials and Methods

### 2.1. Non-Fluorescent Nanoparticles Preparation

The nanoparticles were prepared according to our published protocol [14]. In brief, a solution of chitosan (Chitosan Low Molecular Weight, Sigma-Aldrich, St. Louis, MO, USA) was mixed dropwise with a solution of sodium tripolyphosphate (TPP; Sigma-Aldrich) with or without P10 peptide (GenOne Biotech, Rio de Janeiro, RJ, Brazil). The first solution was composed of 2 mg/mL of chitosan prepared by dissolving the chitosan with 1% of acetic acid (final concentration) in ultrapure water under magnetic stirring for 1 h at room temperature. The pH of the chitosan solution was then adjusted to approximately 4.4 using NaOH 0.1 M. The second solution was composed of 1 mg/mL of TPP and 5 µg (final mass) of the solubilized P10 peptide for the complexed nanoparticles, or without the P10 peptide for the empty nanoparticles.

The second solution (TPP + P10 or TPP) was mixed dropwise in the first solution under magnetic stirring for 90 min at room temperature. After the nanoparticle formation, the nanoparticles were washed with ultra-pure water and centrifuged at 13,200 rpm, 4 °C for 1 h. The washing and centrifugation steps were performed three times. The nanoparticles were then suspended in ultra-pure water for characterization, and then in PBS (vaccine vehicle) or DMEM media (LGC Biotecnologia, Cotia, São Paulo, Brazil) (phagocytosis assay).

### 2.2. Preparation of Fluorescent Nanoparticles

To produce fluorescent nanoparticles, the chitosan was conjugated with the fluorochromes FITC (Sigma-Aldrich) or Cy5.5 NHS ester (Abcam, Cambridge Biomedical Campus, Cambridge, UK) using a protocol adapted from Kwangmeyung Kim, 2010 and Min Huang, 2002 [27,28]. For the FITC conjugation, 15 mg of the fluorochrome was dissolved in 15 mL of DMSO and the chitosan was solubilized with 1% of acetic acid (final concentration) in ultrapure water under magnetic stirring for 1 h at room temperature. The pH of the chitosan was then adjusted to approximately 4.4 using NaOH 0.1 M. The fluorochrome and chitosan solution were then mixed under magnetic stirring for 3–4 h at room temperature or overnight at 4 °C. After stirring, the fluorescent chitosan was precipitated by increasing the pH to 10 with NaOH 0.5 M. For the Cy5.5 conjugation, 1 mg of the fluorochrome was dissolved in 1.5 mL of DMSO, the chitosan was placed in 15 mL of ultra-pure water, and the pH was adjusted to 8.3–8.5 with NaHCO_3_. The fluorochrome and chitosan solution were then mixed under magnetic stirring for 3–4 h at room temperature or overnight at 4 °C. Both the FITC and Cy5.5 fluorescent chitosan were dialyzed for 3 days using a 10 kDa membrane and then lyophilized in the dark until used.

### 2.3. Physical-Chemical Characterization of the Nanoparticles

The sizes of the nanoparticles and polydispersity indexes (PDI) were determined by dynamic light scattering and Zeta Potential (Z potential) was measured by capillary electrophoresis using a Zetasizer nano Zs equipment (Malvern Panalytical Ltd., UK).

### 2.4. Animal Approvals

Pathogen-free male BALB/c nude mice and BALB/c mice, aged 6–8 weeks, were used according to the approval of the ethics committee of the University of São Paulo (CEUA ICB n ° 3654290618).

### 2.5. Experimental Design Used in the Biodistribution Protocol

BALB/c nude mice were randomly organized in the following groups: (C+)—one mouse was inoculated with the fluorochrome only (Cy5.5); (C−)—one mouse was inoculated with PBS; and (NP)—three mice were inoculated with the fluorescent nanoparticles (15 animals in total were utilized).

The BALB/c mice were similarly randomly assigned in the following groups: (C+)—one mouse was inoculated with the fluorochrome only (FITC); (C−)—one mouse was inoculated with PBS; and (NP)—three mice were inoculated with the fluorescent nanoparticles. Lungs, trachea, stomach, bowels and brain were collected (75 animals in total were utilized).

The BALB/c nude and BALB/c mice were anesthetized intraperitoneally with 200 μL of 80 mg kg^–1^ of ketamine and 10 mg kg^−1^ of xylazine prior the administration of 5 μL in each nostril (10 μL in total volume) according to the groups described. The fluorescent Cy5.5 nanoparticles were used to visualize the in vivo biodistribution in the BALB/c nude mice and to evaluate the presence of the nanoparticles in the organs of the BALB/c animals. Images were obtained with the IVIS Spectrum (PerkinElmer, Waltham, MA, USA) located on the Research Facility Center (CEFAP—Institute of Biomedical Science IV, University of São Paulo, São Paulo, SP, Brazil). The animals, or the organs, were analyzed at different times (0, 24, 48, 72 and 96 h) after the inoculation of the fluorescent chitosan nanoparticles.

### 2.6. Nanoparticles Phagocytosis Assay

Alveolar and peritoneal macrophages were collected from non-inoculated mice following a protocol modified from Herb, M. 2019; Zhang, X. 2008; and Busch, C. 2019 [29,30,31]. Alveolar macrophages were collected using BAL (bronchoalveolar lavage) buffer composed of PBS, EDTA 2 mM (final concentration) and 0.5% Fetal Bovine Serum (FBS, Gibco, Thermo Fisher Scientific, Waltham, MA, USA). The BAL buffer and the collected cells were maintained at 37 °C. The peritoneal macrophages were collected and maintained using cold PBS. After collection, both cell types were centrifuged at 3000 rpm at 4 °C for 5 min and seeded in 6-well plates with round glass coverslips at the bottom. The alveolar macrophages were maintained with DMEM supplemented with 10% FBS, 1% (vol/vol) penicillin/streptomycin (pen/strp, Gibco, Thermo Fisher Scientific), 30 ng of GM—CSF (Thermo Fisher Scientific) and 2% pyruvate (Thermo Fisher Scientific). The peritoneal cells were maintained with DMEM supplemented with 10% FBS, 1% (vol/vol) pen/strp and 2% pyruvate (Thermo Fisher Scientific). Both cell types were maintained in cell incubators at 37 °C and 5% CO_2_ until they produced full cell confluence over the coverslips.

The cells were grown in 6-wells plate, each time point in one well. The nanoparticles were added at the same time (0 h).

After full cell confluence of the coverslips, the FITC fluorescent nanoparticles were centrifuged, suspended in 1 mL of DMEM, and added to the cells. The cells were analyzed at different times (0, 2, 4, 6 and 8 h) after the addition of the fluorescent chitosan nanoparticles. Lysosomal Staining Kit—Red Fluorescence (RFP)—Cytopainter (Abcam) was used to provide co-localization of the nanoparticles and the DAPI fluorochrome (Sigma-Aldrich) was used to stain the nucleus. The cells were visualized using a mounting media of 100 μL of 10 × PBS with 900 μL of glycerol in the EVOS Cell Imaging System (Thermo Fisher Scientific).

### 2.7. Yeast Cells

The *P. brasiliensis* strain Pb 18 was maintained as yeast cells in Fava Netto solid media [32]. The yeast cells were transferred to liquid BHI medium (Brain Heart Infusion, Bacto^TM^, BD, Franklin Lakes, NJ, USA) supplemented with 4% FBS, 4% glucose (Difico^TM^, BD) and cultivated at 37 °C and 150 rpm for five to seven days prior to infection. The yeast cells were collected and washed three times using PBS, then centrifuged at 3000 rpm for ten minutes. The yeast cells were suspended in 5 mL of PBS and the viability of the fungal cells was assessed by counting the yeast cells in a Neubauer’s chamber using trypan blue (Gibco, Thermo Fisher Scientific).

### 2.8. Experimental Design Used in the Infection Protocol

BALB/c mice (ten animals per group) were randomly organized in following groups for the two different protocols. For the assessment of activity between the P10 complexed chitosan nanoparticle and the P10 co-administered with the empty particle: (a) Pb 18—positive control, infected without treatment. (b) Associated nanoparticles (Ncomp)—infected and treated weekly with three doses of empty nanoparticles co-administered with 5 μg/10 μL of P10 peptide. (c) Complexed (Comp)—infected and treated weekly with three doses of nanoparticles complexed with 5 μg/10 μL of P10 peptide. (d) SHAM—negative control, uninfected and untreated. 120 animals in total were utilized.

For the experiment testing differences between a single or multiple treatments with P10 complexed with chitosan nanoparticles: (a) Pb 18—positive control, infected without treatment. (b) 1D—infected and treated with one dose of the nanoparticles complexed with 5 μg/10 μL of P10 peptide. (c) 2D)– infected and treated with two doses of the nanoparticles complexed with 5 μg/10 μL of P10 peptide. (d) 3D—infected and treated with three doses of the nanoparticles complexed with 5 μg/10 μL of P10 peptide. (e) SHAM—negative control, uninfected and untreated. Although Comp and 3D are the same regimen, they are named differently for clarity within the different experimental approaches. 150 animals in total were utilized.

### 2.9. Intratracheal Infection and Immunization

To simulate a natural infection caused by the *P. brasiliensis*, BALB/c mice were intratracheally infected with Pb 18 yeast cells at a concentration of 3 × 10^5^ yeast/50 μL. The animals were anesthetized intraperitoneally with 200 μL of 80 mg kg^−1^ of ketamine and 10 mg kg^−1^ of xylazine, an incision was made in the trachea, and 50 μL of the yeast cells suspension was inoculated [14]. After infection, the incision was sutured, and the mice observed and kept warm until complete recovery was documented.

Thirty days after infection, the nanoparticle vaccines were administered to the mice intranasally. Five microliters of the specific formulation were administered into each nostril. For mice receiving more than a single inoculation, the nanoparticles were administered once per week.

### 2.10. Antifungal and Cytokine Evaluation

To determine the effects of vaccination, mice were euthanized 51 days after infection and the lungs were removed. The lungs were macerated manually in 2 mL of PBS, and 100 μL of the tissue was plated on BHI solid media, supplemented with 4% (vol/vol) FBS, 4% (vol/vol) Glucose and 1% (vol/vol) pen/strp. The plates were incubated at 37 °C and the CFU/g of lung were counted 21 days later.

For evaluation of cytokines, 500 μL of the lung macerates were aliquoted into microtubes that contained 500 μL of cOmplete™ protease inhibitor (Roche, Switzerland). Cytokine analysis was performed by Enzyme-Linked Immunosorbent Assay (ELISA) using commercial kits for the following cytokines IL-2, IL- 4, IL-10, IL-12, and IFN-γ (BD OptEIA™, BD), and IL-1β and IL-23 Enzyme-Linked Immunosorbent Assays (ELISA) (Thermo Fisher Scientific). The ODs were assessed using the Epoch 2 Microplate Spectrophotometer (BioTek Instruments, Inc. Winooski, Vermont, EUA).

### 2.11. Statistical Analysis

Analysis of variance (ANOVA) or Student’s *t* test were performed followed by Tukey or Dunnett post-tests using Graph Pad Prism 8. *P* values were considered significant when <0.05. Error bars were expressed as the standard error of the mean (SEM). All experiments were performed in triplicates.

## 3. Results

### 3.1. Nanoparticles Preparation and Characterization

The diameter and size distribution as well as the Z potential of the non-fluorescent nanoparticles presented the desired qualities expected for this type of polymer such that the sizes of the empty nanoparticles were around 230 nm with a PDI less <0.5, and the complexed nanoparticles around 330 nm, also with a PDI <0.5. Similarly, the fluorochrome conjugated nanoparticles complexed with P10 were around 350 nm with a PDI < 0.5. The Z potential was around +20 mV for all particles tested.

### 3.2. Biodistribution using the IVIS Spectrum

**Figure 1:**
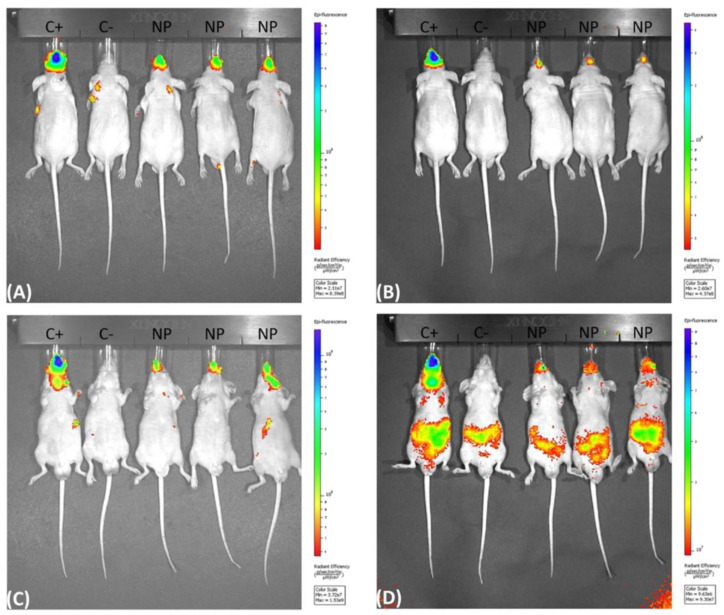
IVIS spectrum fluorescence obtained with the mice positioned in the prone (**A**,**B**) and supine (**C**,**D**) position, showing the fluorescence in the upper air way of the BABL/c nude mice after inoculation of 5 µL per nostril of the fluorescent Cy5.5 chitosan nanoparticles at 0 h (**A**,**C**) and at 96 h (**B**,**D**). (C+) inoculated with the fluorochrome only (Cy5.5), (C−) inoculated with PBS and (NP) inoculated with the fluorescent nanoparticles.

**Figure 2:**
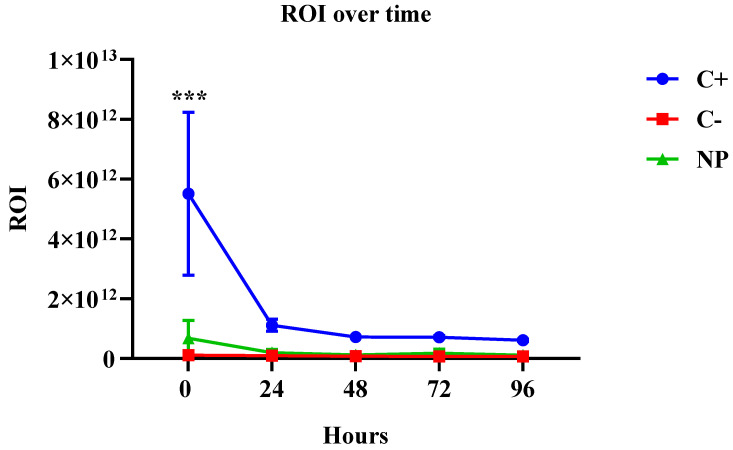
Fluorescent intensity of the region of interest (ROI) over time, showing that the fluorescence was stable during all time points for the NP group, and a significant reduction of the fluorescence for the C+ group in the first 24 h *p* < 0.001.

The fluorescence biodistribution of the Cy5.5-labeled nanoparticles after administration to the BALB/c nude mice were followed for 96 h (5 days; 0 h and 96 h are shown). The IVIS spectrum images were obtained both with the mice positioned in the prone position (Figure 1A,B) and supine position (Figure 1C,D (individual figures can be found in the Supplementary material). A decrease in the fluorescent area was observed over time, suggesting that the nanoparticles were phagocytosed and processed over the interval of 0 and 96 h. By using the IVIS Spectrum to visualize the fluorescent Cy5.5 nanoparticles, we determined that the nanoparticles remained mainly in the upper airway and showed a stable fluorescent intensity at all of the time points, which was different from the control in which there was a significant loss of fluorescent intensity of the region of interest (ROI) in the first 24 h (Figure 2) and (Table 1).

To analyze fluorescence in the organs of the mice, the nanoparticle inoculum had to be concentrated three times (the inoculated volume was not altered). The fluorescence was followed for 96 h (5 days; 0 h and 96 h are shown). Prior to image acquisition, mice were euthanized, and their organs were harvested at each time point. Some of the nanoparticles were present in the trachea and lungs of the mice (Figure S1), but their fluorescence was reduced over time (Figure S2). Stomach and bowels presented background noise fluorescence demonstrated by the fluorescence in the C− group (Figures S3 and S4).

The fluorescence signals in the stomach and bowels of the mice can be explained by the autofluorescence in the mice food, which is well established in the literature [33,34,35,36,37].

In the control animal (PBS), no fluorescence was observed in the upper airway area but we observed fluorescence in the stomach of the animal, although they received PBS only. In the Supplementary material (Figure S3) it is possible to observe stomach and bowels fluorescence in the PBS group from 0 h, and in all mice before the inoculation of any substance (Figure S5).

### 3.3. Nanoparticles Phagocytosis Assay

**Figure 3:**
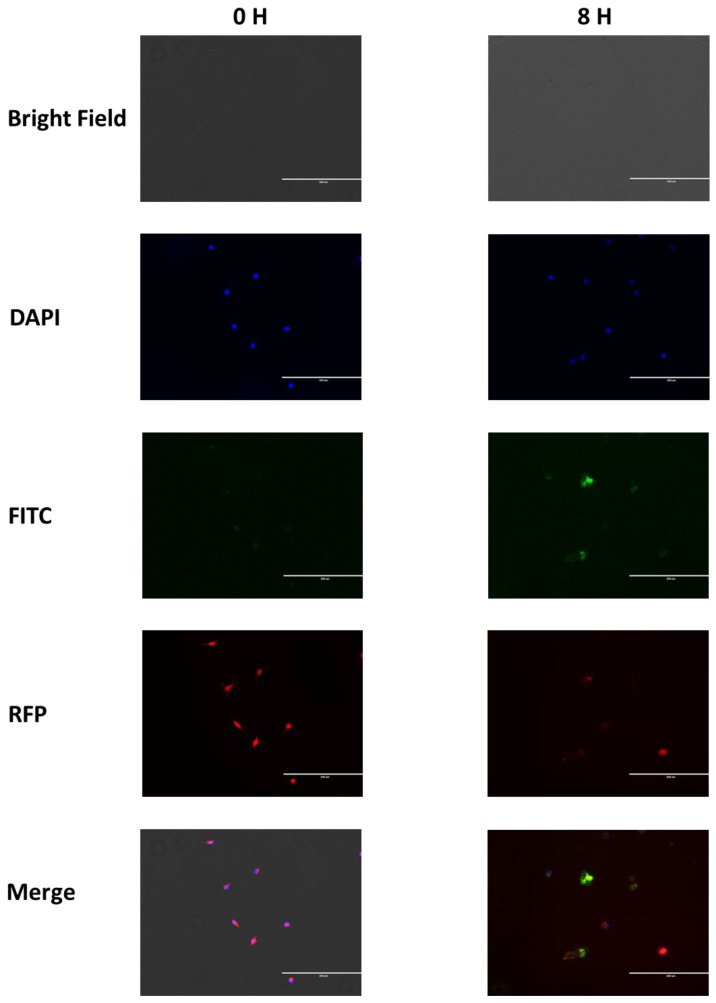
Alveolar macrophage phagocytosis of FITC chitosan nanoparticles at 0 and 8 h showing the nanoparticles inside of cell lysosomes at 8 h. The DAPI filter shows the nuclei, the FITC filter demonstrates the nanoparticles, and the RFP filter indicates the lysosomes.

**Figure 4:**
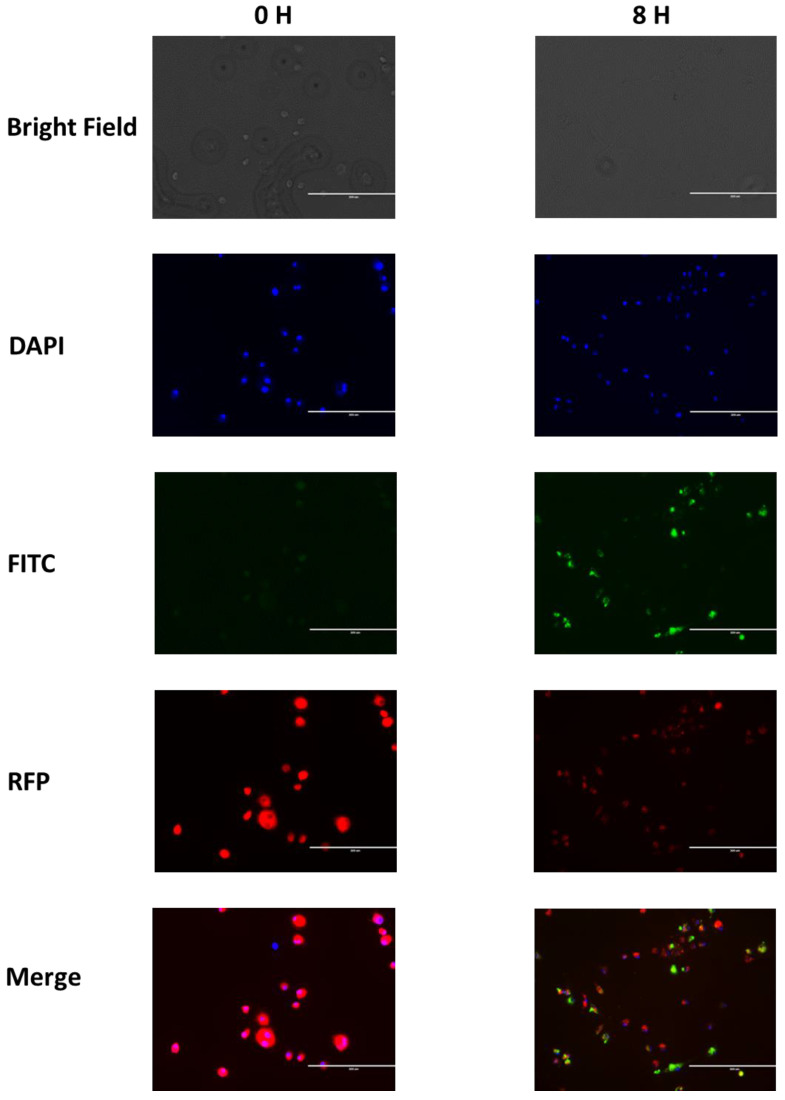
Peritoneal macrophage phagocytosis of FITC chitosan nanoparticles at 0 and 8 h showing the nanoparticles inside of cell lysosomes at 8 h. The DAPI filter shows the nuclei, the FITC filter demonstrates the nanoparticles, and the RFP filter indicates the lysosomes.

The FITC labeled chitosan nanoparticles were phagocytosed by alveolar (Figure 3) and peritoneal (Figure 4) macrophages by 6 h (data not shown) and the maximal phagocytosis occurred by 8 h.

### 3.4. CFU from the Complexed or Co-Administered Nanoparticle Immunizations

**Figure 5:**
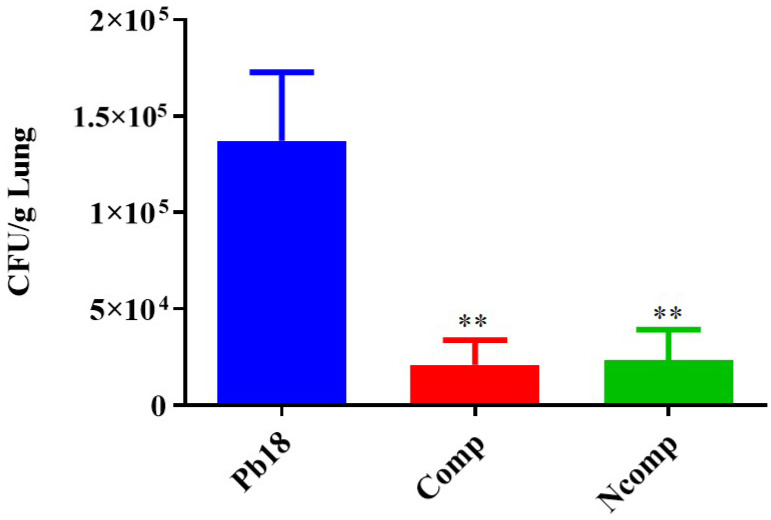
CFU of mice 51 days post infection. The nanoparticle-treated animals had a significant reduction in viable Paracoccidioides cells compared to the Pb 18 group. Pb18 (infected and non-treated), Comp (infected and treated with the P10 complexed nanoparticles) and Ncomp (infected and treated with P10 and with empty nanoparticles). ** = *p* < 0.01.

The animals were euthanized after 51 days of infection during which they were untreated for the first 30 days (establishment of the disease) and then treated with nanoparticles or left untreated (Pb 18, control infected). Then, their lungs were aseptically collected, macerated and aliquots plated to evaluate the efficacy of chitosan nanoparticles complexed with P10 peptide and the adjuvant effect of empty chitosan nanoparticles co-administered with the P10 peptide. The lungs of infected animals in groups Pb18, Comp and Ncomp were significantly heavier than the lungs of control animals (SHAM, not infected and not treated), indicating the success of infection (Figure S6). The CFU determinations showed a significant decrease in viable *Paracoccidioides* cells from the lungs of both groups of nanoparticle-treated mice compared with the untreated group (Figure 5). The CFUs were similar between the P10 complexed nanoparticle-treated mice and the animals that received empty chitosan nanoparticles co-administered with P10.

Our intention was to verify if the immune response was generated by the complexation of the peptide, and if the chitosan nanoparticles possess an adjuvant effect, since the chitosan itself does not possess immunostimulatory effects alone, as with the peptide. In combination, the chitosan and the peptide (complexed or not) were phagocytized by macrophages and dendritic cells and stimulated an Th1 and Th17 cells immune response.

### 3.5. Cytokines from the Complexed or Co-Administered Nanoparticle Immunizations

**Figure 6:**
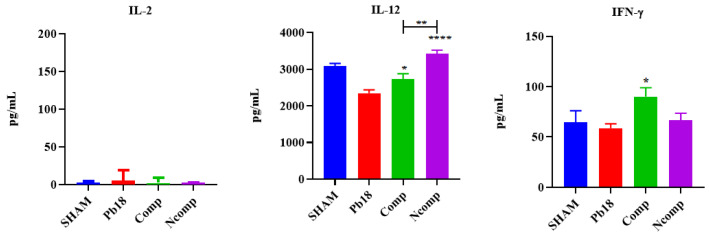
Th1 cytokine production 51 days post infection. No IL-2 was detected, and the IL-12 and IFN-γ were significantly increased, when compared to the Pb18 group. SHAM (non-infected and non-treated), Pb18 (infected and non-treated), Comp (infected and treated with the P10 complexed nanoparticles) and Ncomp (infected and treated with the P10 co-administered with empty nanoparticles). * = *p* < 0.05; ** = *p* < 0.01 and **** = *p* < 0.0001.

**Figure 7:**
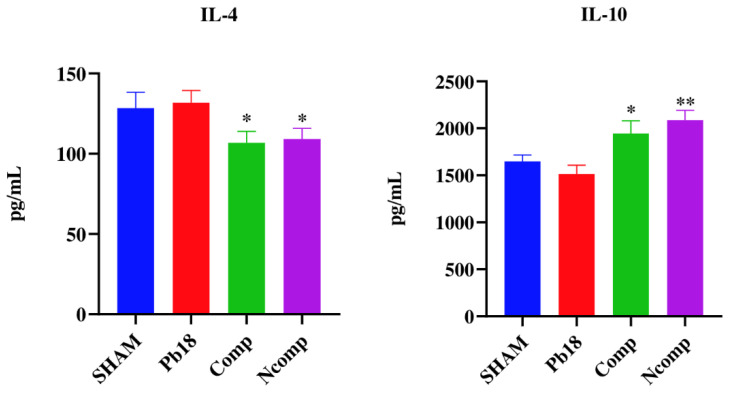
Th2 cytokine production 51 days post infection. IL-4 was significantly decreased, and the IL-10 was significantly increased, for both nanoparticles treated groups when compared to the Pb18 group. SHAM (non-infected and non-treated), Pb18 (infected and non-treated), Comp (infected and treated with the P10 complexed nanoparticles) and Ncomp (infected and treated with the P10 co-administered with empty nanoparticles). * = *p* < 0.05 and ** = *p* < 0.01.

**Figure 8:**
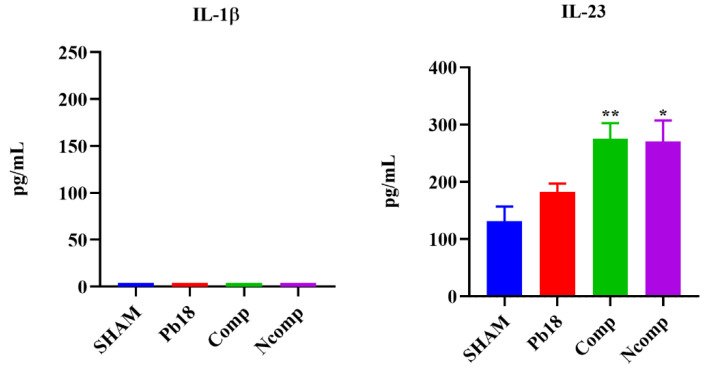
Th17-induced cytokine production 51 days post infection. No changes in IL-1β levels were detected, but IL-23 was significantly increased for both nanoparticles treatment groups when compared to the Pb18 group. SHAM (non-infected and non-treated), Pb18 (infected and non-treated), Comp (infected and treated with the P10 complexed nanoparticles) and Ncomp (infected and treated with the P10 co-administered with empty nanoparticles). * = *p* < 0.05 and ** = *p* < 0.01.

Cytokine production was evaluated 51 days post infection using the same animals as studied for CFUs. The lung macerates from these mice were stored in a −20 °C freezer in microtubes that contained a proteinase inhibitor until they were used for cytokine analysis by ELISA. We focused our evaluation on cytokines associated with the Th1 (Figure 6), Th2 (Figure 7) and Th17 (Figure 8) T cells subset population. We determined that the nanoparticles induced Th1 and Th17 activation, although there was also a small, but not absent, Th2 response.

No IL-2 was detected, and the IL-12 and IFN-γ were significantly increased, when compared to the Pb18 group. IL-12 was higher in the Ncomp group when compared with the Comp group. IL-4 was significantly decreased, and the IL-10 was significantly increased, for both nanoparticle-treated groups when compared to the Pb18 group. No changes in IL-1β levels were detected, but IL-23 was significantly increased for both nanoparticles treatment groups when compared to the Pb18 group.

### 3.6. CFU from the Immunization with Different Doses of Nanoparticles

**Figure 9:**
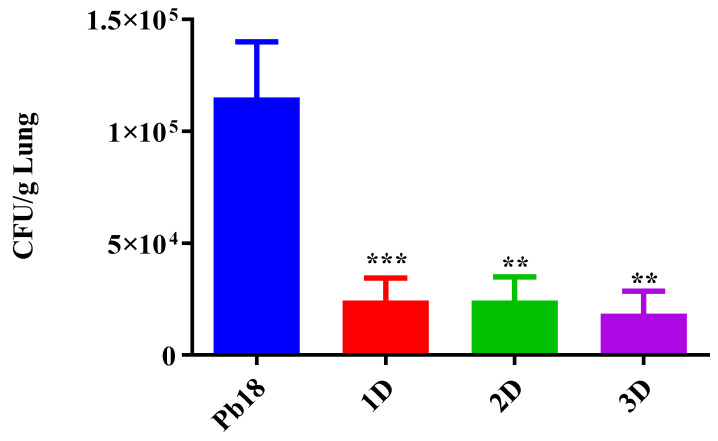
CFU of the lungs 51 days post infection. The P10 complexed nanoparticles significantly reduced the number of the viable Paracoccidioides cells when compared to the Pb 18 group. Pb18 (infected and non-treated), 1D (infected and treated with one dose of the P10 complexed nanoparticles), 2D (infected and treated with two doses) and 3D (infected and treated with three doses). ** = *p* < 0.01 and *** = *p* < 0.001.

The lungs of infected animals (Pb18, 1D, 2D and 3D) were significantly heavier than the lungs of mice from the non-infected and untreated group (SHAM) (Figure S7), showing a successfully infection of the animals by the intratracheal route. The CFUs were significantly lower in the P10 complexed nanoparticle treated animals compared to the infected and untreated mice (Figure 9). Notably, there were no differences between a single treatment with the nanoparticles and either a second or third administration of the compounds one week apart.

### 3.7. Cytokines from the Immunization with Different Doses of Nanoparticles

Based on the reduction of CFUs with only one dose, the pulmonary cytokine production was evaluated in the P18, Sham and 1D groups for responses associated with the Th1, Th2 and Th17 T cells subset population (Table 2). We identified a Th1 and Th17 activation together with a small, but not absent, Th2 response.

No IL-2 was detected, and the IL-12 were significantly increased in the 1D, when compared to the Pb18 group. The IFN-γ were significantly increased in the 1D when compared to the Pb18 group. IL-4 and IL-10 were significantly increased in the 1D group when compared to the Pb18 group. No significant change was observed for the IL-1β, and the IL-23 was significantly increased in all the different doses groups, when compared to the Pb18 group.

## 4. Discussion

PCM is one of the most prevalent yet neglected fungal disease in Latin America, especially in Brazil. New forms of treatment are an important issue since, due to costs and toxicity, the abandonment of treatment and the resurgence of infection is common [3]. In addition to traditional antifungal drugs, novel approaches for preventing or treating fungal diseases have focused on the induction of specific cellular responses that have been demonstrated as ideal for the elimination or containment of fungal infections [10,38]. Our group has successfully demonstrated that the use of polymeric nanoparticles is advantageous for treating PCM disease, and is an exciting new approach to improving current treatment regimens [12,14]. The use of nanoparticles allows for the reduction of the load of the active molecule while serving as adjuvants in vaccines, which can lead to an increase in the cost-effectiveness and efficacy of a vaccine [12,14].

The P10 peptide has been studied for several decades, and its immunomodulatory effect is well described, but its use as a therapeutic has not been optimized as there has been a requirement for large concentrations of the peptide and strong adjuvants, which can sometimes interfere with the modulation of the immune response, to generate a protective host response to PCM disease [12,14,39]. We demonstrated in our studies with nanoparticles that this type of delivery platform can reduce the amount needed to effectively modify the immune response to the benefit of the host [12,14].

To further optimize the delivery of the P10 peptide using chitosan nanoparticles, we performed biodistribution assays. We observed that the nanoparticles were phagocytosed in the first 4 to 6 h of interaction with the cells, and that most of the nanoparticles adhered to the mucosa of the upper respiratory tract; smaller amounts were detected in the trachea and lung where they remained for at least one week. These findings are consistent with characteristics conferred by chitosan nanoparticles due to their mucoadhesive capacity [24,40,41].

IVIS experiments are complicated by the background fluorescence in the stomach and bowels of the mice. This background fluorescence is a consequence of the presence of chlorophyll-containing alfalfa and its derivates in mouse food. These substances present autofluorescence in the same frequencies of excitation and emission of near infrared fluorochromes [33,34,35,36,37]. For compliance with animal welfare requirements, the mice in our experiments received food and water ad libitum.

As demonstrated by different researchers, the volume of inoculation, anesthesia induction status and position of the animal during and after vaccination, are factors that may affect the correct localization of the vaccines based in nanoparticles or not after intranasal administration [42,43,44].

In our experimental design we tried to reduce as much as possible the variables that could influence the correct location of the nanoparticles, since the animals’ food presented fluorescence similar to that of the fluorochrome used. Even with these precautions, we cannot rule out the possibility that a small amount of the vaccine could have been swallowed, even though it was designed for degradation in the airway mucosa and can be partially degraded in the low pH of the stomach or peptidases in the intestinal tract [33,34,35,36,37,42,43,44].

The reduction of the fungal load in infected mice treated with P10 complexed to the nanoparticles was expected based on previous work from our research group using chitosan nanoparticles as an intranasal vaccine to treat PCM disease [14]. The innovation of this new work was the reduction of the number of doses and the reduction of the concentration of the P10 peptide when co-administered with empty chitosan nanoparticles, demonstrating the adjuvant and immunomodulatory effects of the chitosan nanoparticles [45,46,47,48,49,50,51].

The reduction of the necessary vaccine doses and the P10 concentration are directly related to a reduction of cost. Here, we show the reduction of the total amount of P10 peptide is usually used as standard (60 µg in three doses) to 12 times less (5 µg in one single dose) for the complexed nanoparticles, and to 4 times less when the peptide co-administrated with the empty nanoparticles [1,12,14].

The adjuvant effect of chitosan makes it an ideal choice for use in vaccines that aim to induce immune responses with an emphasis on cellular and non-humoral recruitment [45,46,47,48,49,50,51].

In our previous work we demonstrated that the chitosan nanoparticles alone had no immunomodulatory effect [14].

Chitosan stimulates phagocytic cells such as macrophages and dendritic cells to produce type 1 interferons and interleukins, especially IL-1β, and this stimulation is based on the interaction of chitosan with TLR-4 receptors, stimulation of the NLRP3 inflammasome (necessary for combating and or containing *Paracoccidioides* [6]), and activation of cell signaling pathways (STING: stimulator of interferon genes) that function as a nucleic acid detector, and cGAS (guanine and adenine cyclic dinucleotides synthase) that is a precursor and co-stimulator of STING [45,46,47,48,49,50,51].

Mucosal immunity is another key factor related to the reduction of the fungal load enhanced by the adjuvant effect of the chitosan nanoparticles [52,53]. The upper airway mucosa is known as one of the main factors related to prevent infections [54]. The nasal region has different mechanisms related to innate and adaptative immunity, such as the mucus itself, which is full of enzymes and proteins related to degradation, inactivation and opsonization of dangerous particles or microorganisms [19]. Phagocytic cells and antigen present cells are also found in the mucosa of the upper airway, and these cells may be responsible for the uptake of the chitosan nanoparticles [52,53,55]. After uptake, the adjuvant effect of the chitosan is activated leading to the stimulation or restimulation of the effector mechanism responsible to contain or eliminate the *Paracoccidioides* in the lungs [56,57,58]. Due to the expected nanoparticle-induced immune response, and the type of immune response elicited by the infection, we analyzed the group of cytokines mainly associated with a protective response to fight fungal infections. These cytokines belong to Th1 and Th17 cell subsets, which are the responsible for coordinating the clearance of PCM disease [6].

The complexed vs co-administered formulation experiment was designed to verify if the chitosan nanoparticles act as an adjuvant when utilized without complexation with the peptide (co-administrated), and also to validate if this adjuvant effect would work with smaller amounts of the peptide in relation to our previous work [14].

Our model of infection (30 days prior treatment) was designed to simulate the chronic granulomatous PCM [6,7,59,60]. Increase of these cytokines can be observed in the early days of infection, or later during the adaptative immune response to the infection with granuloma formation. After the granuloma formation, the immune response is reduced to prevent tissue damage. This is one of the most remarkable characteristics of PCM [6,7,59,60].

The complexed and co-administrated nanoparticle chitosan vaccines were able to increase the levels of IL-12 and IFN-γ, which are important Th1 cytokines related to inflammation and elimination of the *Paracoccidioides;* however, no IL-2 was detected, probably because its release occurs in the first 24 h of stimulation [6,7,59,60]. The levels of IL-4 were reduced and the levels of IL-10 was slightly increased. These Th2 cytokines are related with inflammation control, associated with tissue repair or cell reorganization, and are important to the regulation of granuloma formation [6,7]. No IL-1β was detected, but the levels of IL-23 were increased, pointing to a possible presence of the Th17 T cell subset [6,7,56].

Similar cytokines results were observed in the single dose vaccine group, showing increased levels of Th1 and Th17-related cytokines and the increased presence of Th2 cytokines. We observed an increase in the IL-12, IFN-γ, IL-4 and IL-10 cytokine levels of the treated group (1D) in relation to the controls groups (SHAM and Pb18), and statistical analysis was performed in comparison with the 1D group and infected with Pb 18 group [6,7,56]. Small variations in cytokine levels were detected between batches of mice, even though they were SPF animals; however, the general pattern of response was similar across the different experiments [6,7,56].

Based on our findings, we concluded that the chitosan nanoparticles adhered to the mucosa were constantly and slowly phagocytosed, inducing the production of inflammatory cytokines type Th1 and Th17, reducing the fungal load in the lungs.

## 5. Conclusion

We demonstrated that our chitosan nanoparticles remained mostly in the upper airway of the mice when administered intranasally, with a smaller amount localizing in the trachea and lungs due to chitosan muco-adhesiveness; the nanoparticles were phagocytized after 4 to 6 h. The chitosan nanoparticles show strong potential as an adjuvant for vaccines effectively inducing Th1 and Th17 immune responses, which are ideal for fighting fungal infections. The nanoparticles also reduced the need for multiple doses to significantly reduce the fungal burdens in the lungs of infected animals.

## 6. Patents

INPI: BR 10 2019 012313 3 A2.

## Figures and Tables

**Table 1:** Fluorescent intensity of the region of interest (ROI) over time, showing the specific average values of the fluorescence intensity for all time points.

	Fluorescent Intensity of the Region of Interest (ROI)		
Hours	C+	C−	NP
0	5.5 × 10^12^	1.0 × 10^11^	6.7 × 10^11^
24	1.1 × 10^12^	9.3 × 10^10^	1.9 × 10^11^
48	7.2 × 10^11^	7.6 × 10^10^	1.1 × 10^11^
72	7.1 × 10^11^	7.3 × 10^10^	1.7 × 10^11^
96	6.0 × 10^11^	7.2 × 10^10^	1.1 × 10^11^

(C+) inoculated with the fluorochrome only (Cy5.5), (C−) inoculated with PBS and (NP) inoculated with the fluorescent nanoparticles.

**Table 2:** Th1, Th2 and Th17 cytokine production 51 days post infection.

	Cytokines						
Groups	Th1			Th2		Th17	
	IL-2	IL-12	IFN-γ	IL-4	IL-10	IL-1β	IL-23
Sham	0	2605 ± *480.1*	80.96 ± *22*	148.4 ± *57.84*	1965 ± *297.8*	0	339.4 ± *208.9*
Pb 18	0	2491 ± *502.8*	66.80 ± *9.9*	140.4 ± *37.61*	2000 ± *387.7*	0	117.0 ± *107.2*
1D	0	3168 ± *438.5* ***	92.84 ± *29.38* *	193.6 ± *47.72* **	2418 ± *299.8* **	0	232.4 ± *87.20* **

Th1, Th2 and Th17 cytokine production when compared to Pb18 group. Numbers in italic = SD, * = *p* < 0.05; ** = *p* < 0.01 and *** = *p* < 0.001.

## Data Availability

Not applicable.

## Supplementary Materials

The following supporting information can be downloaded at: https://www.mdpi.com/article/10.3390/jof9020245/s1, Figure S1: IVIS spectrum fluorescence of lung (L), trachea (T) and stomach (S), after inoculation of 5 µL per nostril of the fluorescent Cy5.5 chitosan nanoparticles (NP) at 0 h; Figure S2: IVIS spectrum fluorescence of lung (L), trachea (T), stomach (S) and brain (B), after inoculation of 5 µL per nostril of the fluorescent Cy5.5 chitosan nanoparticles (NP) at 96 h; Figure S3: IVIS spectrum fluorescence of lung (L), trachea (T), stomach (S), brain (Br), liver (L) and bowels (Bo) after inoculation of 5 µL per nostril of PBS at 0 h; Figure S4: IVIS spectrum fluorescence of lung (L), trachea (T), stomach (S) and brain (Br), after 96 h of the inoculation of 5 µL per nostril of PBS; Figure S5: IVIS spectrum fluorescence obtained with the mice positioned in supine position showing the fluorescence in the gastrointestinal tract of the BABL/c nude mice before the inoculation of any substance; Figure S6: Weight of the lungs after euthanasia (51 days post infection). There was a significant increase of the lung weight from infected animals when compared to the SHAM group. SHAM (non-infected and non-treated), Pb18 (infected and non-treated), Comp (infected and treated with the P10 complexed nanoparticles) and Ncomp (infected and treated with the P10 associated with empty nanoparticles). * = *p* < 0.05 and ** = *p* < 0.01; Figure S7: Weight of the lungs after euthanasia (51 days post infection). There was a significant increase of the lung weight from infected animals, when compared to the SHAM group. SHAM (non-infected and non-treated), Pb18 (infected and non-treated), 1D (infected and treated with one dose of the P10 complexed nanoparticles), 2D (infected and treated with two doses of the P10 complexed nanoparticles) and 3D (infected and treated with three doses of the P10 complexed nanoparticles). * = *p* < 0.05 and ** = *p* < 0.01.
