# Supplementary material for: Biodistribution and Adjuvant Effect of an Intranasal Vaccine Based on Chitosan Nanoparticles against Paracoccidioidomycosis
Source: J Fungi (Basel). 2023 Feb 12;9(2):245. doi: 10.3390/jof9020245 (PMC9964167; doi:10.3390/jof9020245)
Supplement: Supplementary file 1 [file jof-09-00245-s001.zip › jof-2075051-supplementary.pdf]

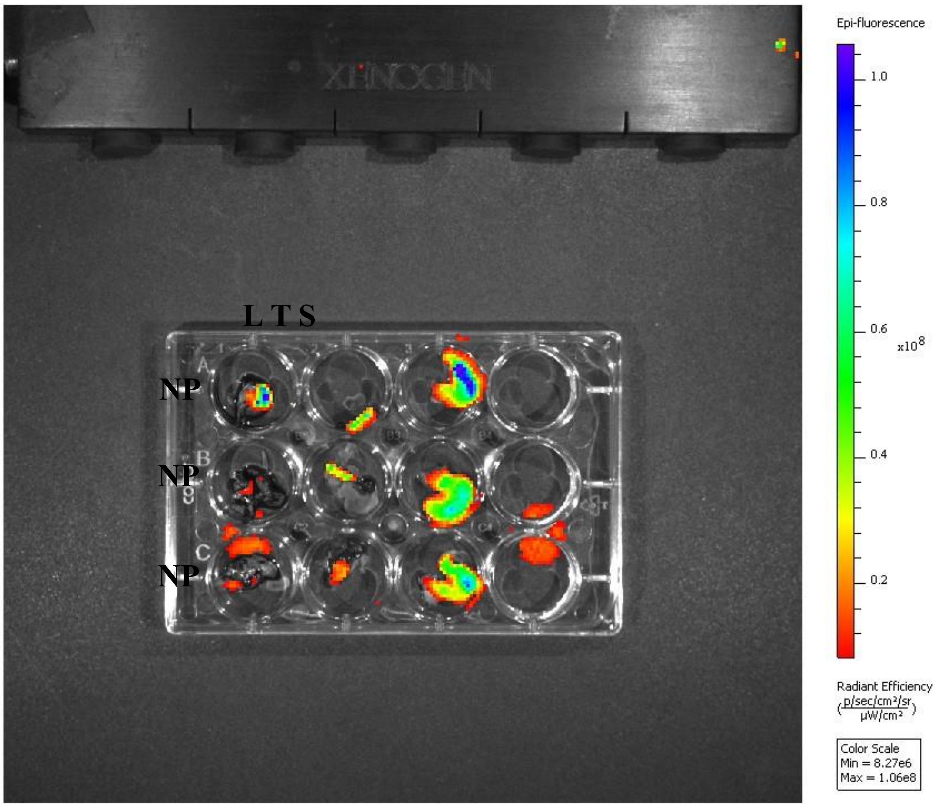

Figure S1. IVIS spectrum fluorescence of lung (L), trachea (T) and stomach (S), after inoculation of 5 μL per nostril of the fluorescent Cy5.5 chitosan nanoparticles (NP) at 0 h.

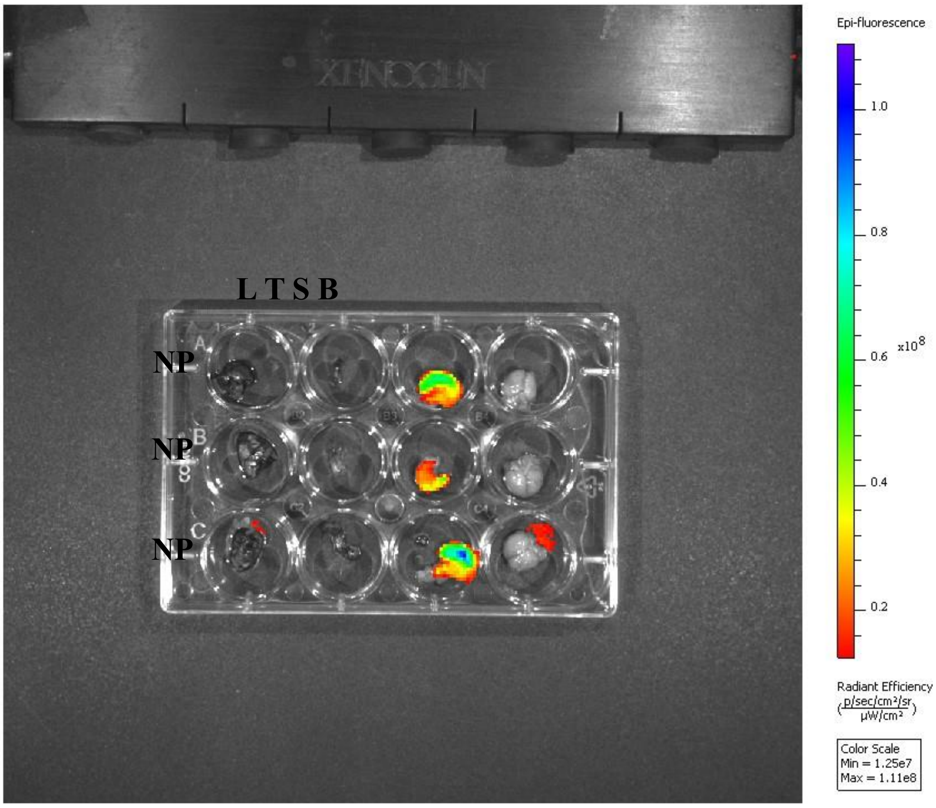

Figure S2. IVIS spectrum fluorescence of lung (L), trachea (T), stomach (S) and brain (B), after inoculation of 5 μL per nostril of the fluorescent Cy5.5 chitosan nanoparticles (NP) at 96 h.

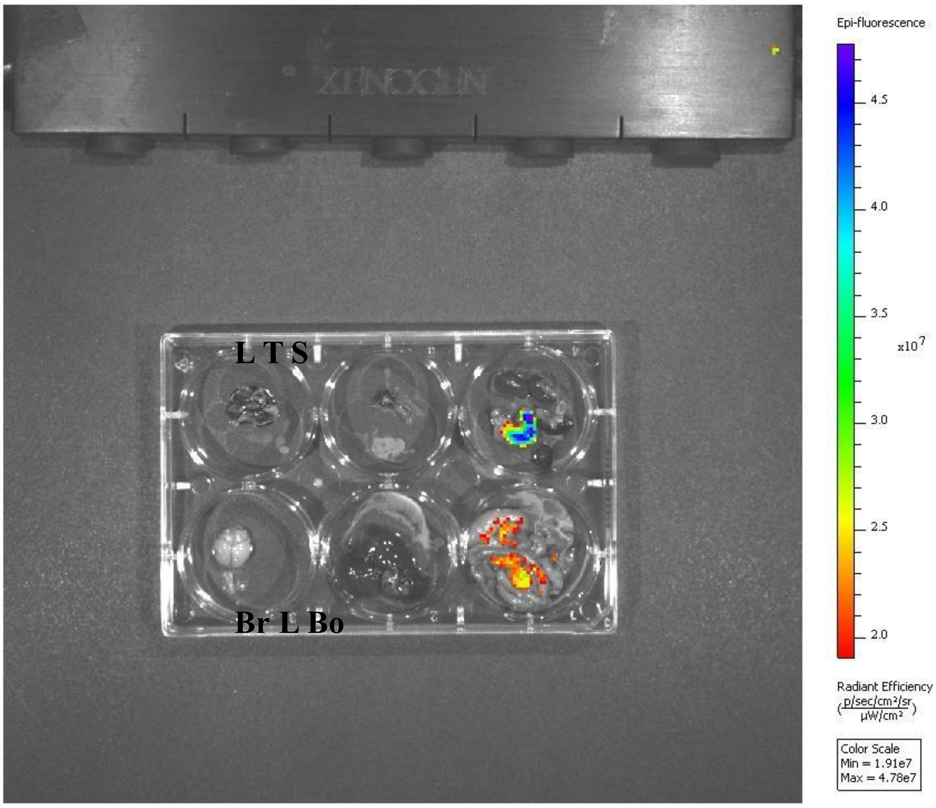

Figure S3. IVIS spectrum fluorescence of lung (L), trachea (T), stomach (S), brain (Br), liver (L) and bowels (Bo) after inoculation of 5 μL per nostril of PBS at 0 h.

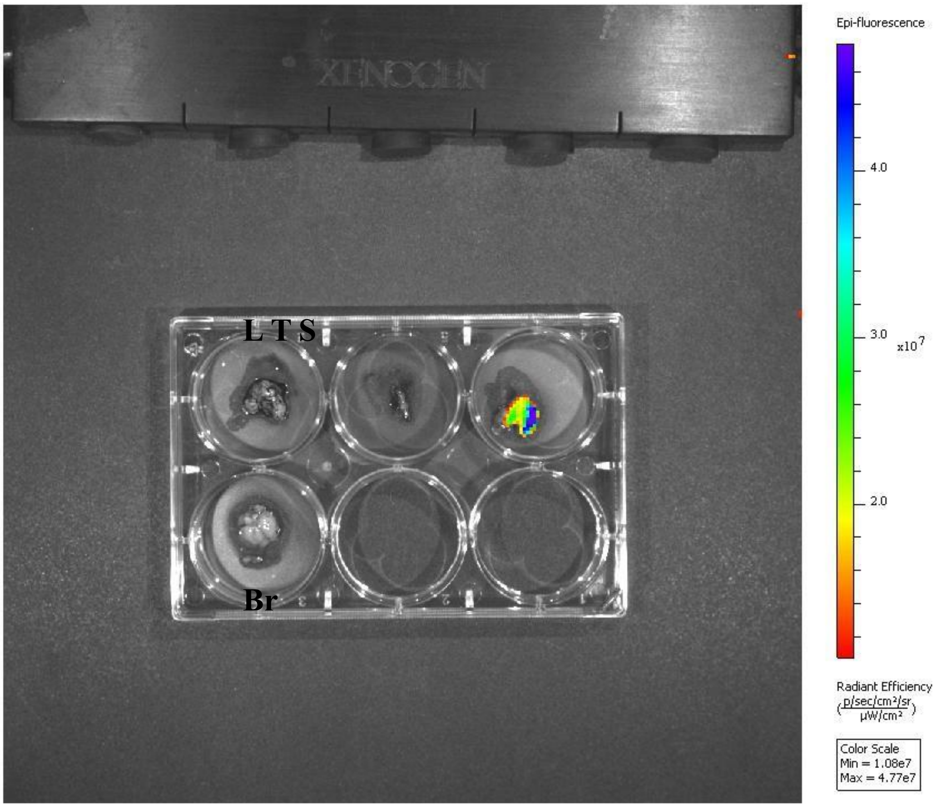

Figure S4. IVIS spectrum fluorescence of lung (L), trachea (T), stomach (S) and brain (Br), after 96 h of the inoculation of 5 μL per nostril of PBS.

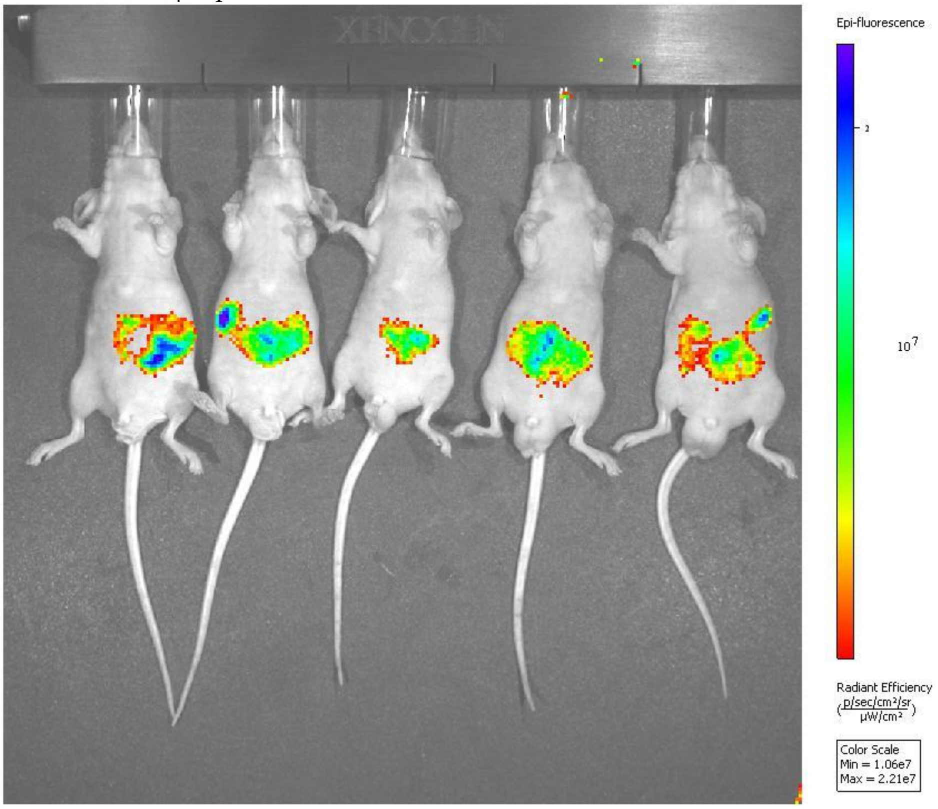

Figure S5. IVIS spectrum fluorescence obtained with the mice positioned in supine position showing the fluorescence in the gastrointestinal tract of the BABL/c nude mice before the inoculation of any substance.

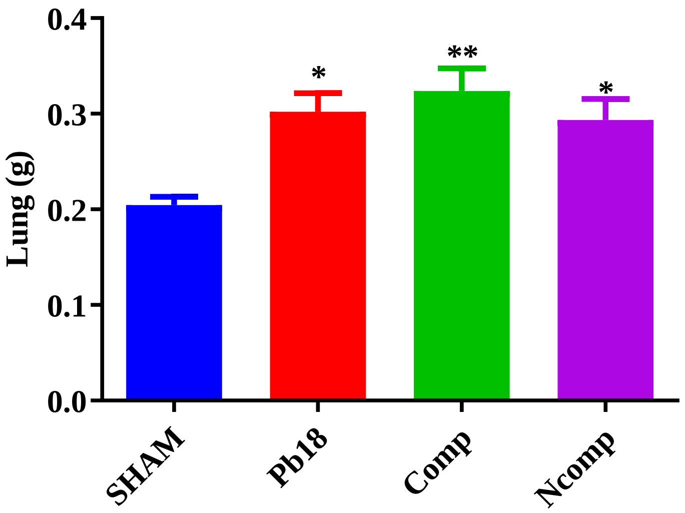

Figure S6. Weight of the lungs after euthanasia (51 days post infection). There was a significant increase of the lung weight from infected animals when compared to the SHAM group. SHAM (non-infected and non-treated), Pb18 (infected and non-treated), Comp (infected and treated with the P10 complexed nanoparticles) and Ncomp (infected and treated with the P10 associated with empty nanoparticles). * = $p < 0.05$ and ** = $p < 0.01$.

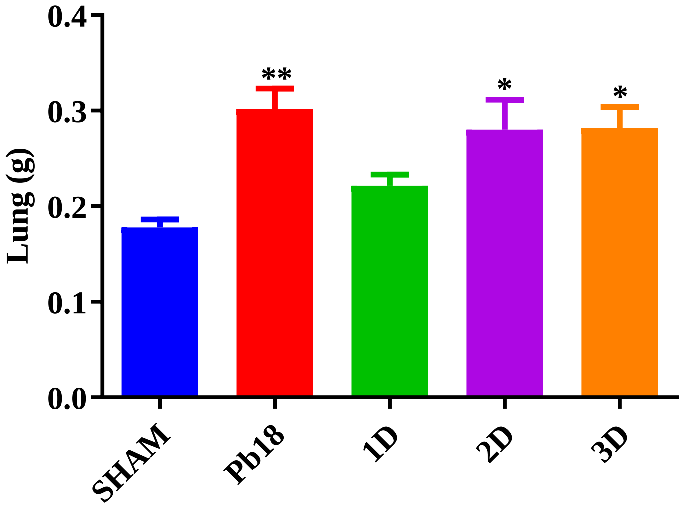

Figure S7. Weight of the lungs after euthanasia (51 days post infection). There was a significant increase of the lung weight from infected animals, when compared to the SHAM group. SHAM (non-infected and non-treated), Pb18 (infected and non-treated), 1D (infected and treated with one dose of the P10 complexed nanoparticles), 2D (infected and treated with two doses of the P10 complexed nanoparticles) and 3D (infected and treated with three doses of the P10 complexed nanoparticles). * = $p < 0.05$ and ** = $p < 0.01$.
